# Differential Development of Human Brain White Matter Tracts

**DOI:** 10.1371/journal.pone.0023437

**Published:** 2011-08-31

**Authors:** Davide Imperati, Stan Colcombe, Clare Kelly, Adriana Di Martino, Juan Zhou, F. Xavier Castellanos, Michael P. Milham

**Affiliations:** 1 Phyllis Green and Randolph Cowen Institute for Pediatric Neuroscience, Child Study Center, Langone Medical Center, New York University, New York City, New York, United States of America; 2 Nathan Kline Institute for Psychiatric Research, Orangeburg, New York, United States of America; Cuban Neuroscience Center, Cuba

## Abstract

Neuroscience is increasingly focusing on developmental factors related to human structural and functional connectivity. Unfortunately, to date, diffusion-based imaging approaches have only contributed modestly to these broad objectives, despite the promise of diffusion-based tractography. Here, we report a novel data-driven approach to detect similarities and differences among white matter tracts with respect to their developmental trajectories, using 64-direction diffusion tensor imaging. Specifically, using a cross-sectional sample comprising 144 healthy individuals (7 to 48 years old), we applied k-means cluster analysis to separate white matter voxels based on their age-related trajectories of fractional anisotropy. Optimal solutions included 5-, 9- and 14-clusters. Our results recapitulate well-established tracts (e.g., internal and external capsule, optic radiations, corpus callosum, cingulum bundle, cerebral peduncles) and subdivisions within tracts (e.g., corpus callosum, internal capsule). For all but one tract identified, age-related trajectories were curvilinear (i.e., inverted ‘U-shape’), with age-related increases during childhood and adolescence followed by decreases in middle adulthood. Identification of peaks in the trajectories suggests that age-related losses in fractional anisotropy occur as early as 23 years of age, with mean onset at 30 years of age. Our findings demonstrate that data-driven analytic techniques may be fruitfully applied to extant diffusion tensor imaging datasets in normative and neuropsychiatric samples.

## Introduction

Throughout the lifespan, the human brain is continuously shaped by genetic and environmental factors, as well as their developmental interplay [1]. Histological studies in human and non-human primates have shown that these complex patterns of age-related changes involve both gray and white matter [2], [3], [4], [5]. In longitudinal studies of human development, high-resolution anatomic MRI studies of cortical thickness as well as gray and white matter volumes have detected complex region-specific maturational trajectories (e.g., quadratic, cubic) *in vivo* [6].

Analyses of white matter development have been enhanced by the availability of diffusion tensor imaging (DTI), which provides information regarding white matter microstructure, including fractional anisotropy (FA) [7]. Studies directly comparing children and adults, and those examining linear age-related trends, have consistently demonstrated developmental increases in FA from childhood into early adulthood [8] [9], [10], [11], [12], [13], [14], [15], [16]. At the other end of the developmental spectrum, age-related decreases in FA have been observed beginning in middle adulthood and accelerating rapidly in later life [10], [11], [12], [13], [14], [15], [16], [17], [18], [19], [20].

This pattern of increasing FA from childhood to adulthood, with subsequent decreases late in life, strongly suggests that white matter maturation follows non-linear trajectories across the lifespan. Many initial efforts to characterize white matter maturation have assumed linear age effects, an assumption that is not necessarily justified and can lead to false negatives as a result of inadequate fitting [19]. More recent developmental studies of FA suggest that exponential models provide a better fit than linear models [18], [20]. However, exponential curves may be suboptimal for appraising lifespan developmental changes in FA, where inverted u-shaped trajectories are expected [17], [18], [19], [20]. Additional challenges arise when attempting to identify differential rates of white matter maturation across different tracts, or across subdivisions within the same tract. One common approach to address this challenge is to define tracts on the basis of previously established probabilistic atlases [20]. Such atlases tend to provide only large-scale tract definitions, thus limiting the resolution at which tracts and their subdivisions can be differentiated in terms of developmental trajectories. Boundary regions between tracts can be another significant source of variation across subjects. To bypass these problems in tract definition, investigators have relied on tractography-based approaches [19]. Yet, tractography is also subject to several sources of error, including the requirement for user-based specification of seed and termination locations and other parameters (e.g., stop length, Euler integration), as well as inadequate spatial resolution, particularly in the vicinity of fiber crossing/kissing [21]. Potentially most problematic is the current paucity of sophisticated approaches for assessing inter-individual and group-related differences in tractography, which limits the utility of tractography approaches for characterizing white matter maturation in the developing brain.

Here, in a cross-sectional sample composed of 144 individuals (7 to 48 years old), we demonstrate an alternative approach for exploring differential developmental trajectories across white matter tracts and their subdivisions that requires no *a priori* information. Specifically, we used k-means cluster analysis [22] to separate white matter voxels based on their age-related trajectories. This approach was grounded in two fundamental principles; 1) that, in a given dataset, a finite number of differential developmental trajectories would be detectable within the central “skeleton” of major white matter tracts [23], and 2) that structurally related voxels would exhibit similar developmental trajectories, and thus cluster together [24], [25]. We employed an established clustering index [26] to identify optimal solutions and avoid over-clustering (i.e., dividing a single population of voxels across multiple clusters). Lower order cluster solutions can reveal large-scale structural systems (i.e., sets of tracts), while higher order solutions reveal individual tracts and their subdivisions (i.e., the higher the number of clusters examined, the more detailed the differentiation), thus permitting a more systematic examination of white matter structures and their development. Finally, to demonstrate the ability of our method to delineate well-established tracts, we sort the findings in terms of the DTI-81 Atlas, which was used as a “gold standard”.

## Materials and Methods

### Participants

This study is based on DTI data obtained from 144 healthy individuals aged 7–48 years (mean age = 20.8±12.0 yrs, 69 males). All participants were free from psychiatric disorders, as confirmed by semi-structured psychiatric interviews. Specifically, absence of DSM-IV axis-I psychiatric disorders was established per the Schedule for Affective Disorders and Schizophrenia for School-Age Children – Present and Lifetime Version (KSADS-PL) [27] administered to parents and their children. The Structural Clinical Interview for DSM-IV-TR Axis I Disorders, Research Version, Non-patient Edition (SCID-I/NP) [28] and the Adult ADHD Clinical Diagnostic Scale (ACDS) V.1.2 [29] were administered to adults (>18 years). Right-handedness, absence of chronic medical conditions, and of contraindications for MRI were required for all participants. The institutional review boards of the New York University School of Medicine and New York University approved this study. Prior to participation, written informed consent and assent (for participants<18 years) were obtained from all participants and their parents/legal guardians (for participants<18 yrs). Participants received monetary compensation for completing the study.

### Acquisition

Imaging data were acquired using a Siemens Allegra 3T (NYU Center for Brain Imaging). A T1-weighted image (MPRAGE, TR = 2530 ms; TE = 3.25 ms; TI = 1100 ms; flip angle = 7°; 128 slices; FOV = 256 mm; voxel-size = 1×1.3×1.3 mm), and two DTI scans were acquired from each participant using a twice-refocused diffusion-weighted EPI sequence (TR = 5200 ms; TE = 78 ms; 50 slices; acquisition matrix 64×64; FOV = 192 mm; acquisition voxel size = 3×3×3 mm; 64 diffusion directions chosen to be uniformly distributed around a unit sphere [30] with b-value 1000 s/mm^2^; 1 image with no diffusion weighting). Bandwidth was 3720 Hz/pixel. A gradient echo field map (TR = 834 ms; TEs = 5.23 and 7.69 ms) was also acquired with the same slice positioning and resolution as the diffusion-weighted data.

### DTI preprocessing

The two diffusion weighted imaging (DWI) datasets for each participant were concatenated. Motion correction was performed by applying 9 degrees of freedom linear registration to a warped template, derived from the field map. Local magnetic field inhomogeneities were accounted for by field map reconstruction [31]. Diffusion gradients were rotated to improve consistency with the motion parameters and data for each of the 128 corrected directions (i.e., from the two 64-direction scans) were used to fit the tensor parameters, to improve signal to noise ratio [30]. Diffusion tensors were fitted for each voxel to obtain FA images, which were registered to the FMRIB58_FA standard space image with 1 mm^3^ resolution using the non-linear registration tool FNIRT [32], [33]. A group-mean FA skeleton was created, and each participant's standard space FA data was projected onto the FA skeleton using the standard preprocessing scripts provided with FSL's Tract-Based Spatial Statistics (TBSS). The resultant skeletonised FA images were used for further analysis.

### Cluster Analysis

Prior to cluster analysis, differences among participants related to registration to standard space were removed from the data by regressing out the mean squared difference between each participant's FA map and the FMRIB58_FA template [34]. Next, the k-means algorithm was used to sort voxels into clusters based upon their zero-centered FA age-related trajectory (i.e., the FA values obtained across participants, corresponding to the measured FA values at 144 points over the age range 7–48 years). The k-means algorithm [22] is a computationally efficient approach that tends to produce tighter clusters than more basic algorithms (e.g., hierarchical clustering [35]), as it optimizes the distance between voxels and cluster centroids. The k-means algorithm has a single free parameter: the number of clusters. Given the lack of *a priori* expectations with respect to the number of clusters, we repeated the algorithm varying the number of clusters from 2 to 16.

A classic challenge for data-driven approaches such as cluster analysis is the determination of an “optimal solution”. A common way to address this challenge is to calculate a measure of cost for each cluster solution, and identify those solutions with local minima in the cost measure as optimal. Here, to assess the stability of each clustering solution, we used the Davies-Bouldin cluster validation index (DBI) [26]. This measure is a ratio of the mean within-cluster distance to the mean distance between cluster centers, weighted by the number of clusters. Solutions that represented local DBI minima were identified as optimal and reported in the present work.

#### Standard Model-Based Analyses

Using FSL 4.1.5 (FMRIB Software Library, Oxford, UK; http://www.fmrib.ox.ac.uk/fsl/) we applied a general linear model to each voxel of raw statistical TBSS images using permutation-based non-parametric testing. We corrected for multiple comparison using threshold-free cluster enhancement (p<0.05, corrected) [36]. To evaluate the improvement of the second order fit over linear fit we fitted linear and quadratic models to the skeleton voxels and carried out an F-test using in-house developed MATLAB (The MathWorks, Inc., Natick, MA) functions.

## Results

### Overview

**Figure 1:**
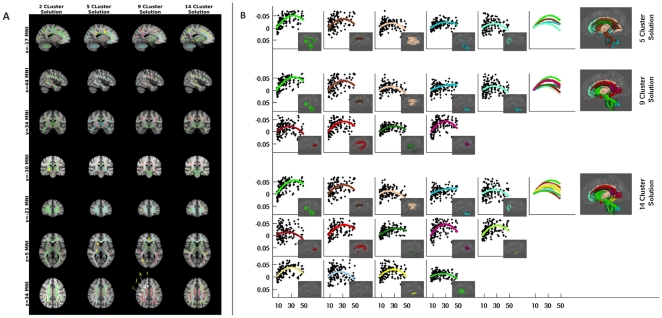
Optimal Cluster Solutions. *Panel A (Skeletonized Fractional Anisotropy [FA])*: The 5-, 9- and 14- cluster solutions identified by the Davies-Bouldin cluster validation index as optimal are depicted here, along with the 2-cluster solution, for reference. Cluster analysis recapitulated well-established white matter tracts, as well as key distinctions within white matter tracts. Notable examples include separation of the corpus callosum (CC) into three major divisions (splenium, body, genu) and differentiation of anterior and posterior limb of the internal capsule. Across all solutions, the cerebral peduncles and anterior limb of the internal capsule demonstrated consistent clustering patterns, likely reflecting fronto-pontine pathways. Tract Marker Key: [A: splenium (CC), B: body (CC), C: genu (CC), D: corticospinal tract (marked at interface with cerebral peduncle), E: anterior limb of the internal capsule, F: posterior limb of the internal capsule, G: forceps minor, H: optic radiations, I: superior longitudinal fasciculus, J: superior corona radiata, K: body (CC), L: cingulum bundle]. *Panel B (Trajectories and Atlas-Based Projections)*: For each of the optimal cluster solutions (5-, 9- and 14-clusters), we depict the mean trajectory across voxels in each of the clusters; all trajectories are baselined with respect to the initial trajectory value to facilitate visual comparison. Values on the ordinates represent change in FA from the initial value obtained for each trajectory for each cluster. Age in years is shown on the abscissas. Across clusters, the mean age at which peak FA was reached was 30.1 years. To facilitate visualization, we provide ICBM-81 atlas-based tract projections, with each tract color-coded based upon the dominant cluster to which its voxels were assigned (note: clusters 11 and 12 were not dominant in any atlas-based tract). Immediately to the left of each anatomic projection is the overlay of the trajectories for each solution.

Cluster analyses grouped and differentiated white matter voxels on the basis of their age-related FA trajectories. The Davies-Bouldin cluster validation index (DBI) suggested that the 5-, 9- and 14- cluster solutions were optimal. The resulting clusters appeared to meaningfully recapitulate well-established white matter tracts and subdivisions within tracts, as well as relationships among tracts. Importantly, across cluster solutions, many clusters exhibited a notable degree of stability (e.g., clusters one and two remained relatively stable across the 3–6 cluster solutions, cluster 3 was relatively stable across the 4–6 cluster solutions, and so on), with changes occurring in a progressive hierarchical manner from one solution to the next (Figures S1a and S1b depict the evolution of the first five cluster solutions). Figures 1a and 1b depict the optimal cluster solutions. Importantly, clusters were generally found to be composed of large continuous tracts (see Table S1 for quantifications of continuity). Lower order cluster solutions tended to combine a larger number of tracts into a single cluster, while higher order solutions better differentiated larger tracts from one another (i.e., separated them into different clusters). Higher order solutions (e.g., 11, 13, 14) tended to have some clusters that comprised smaller collections of continuous voxels, generally located near the gray-white interface.

**Figure 2:**
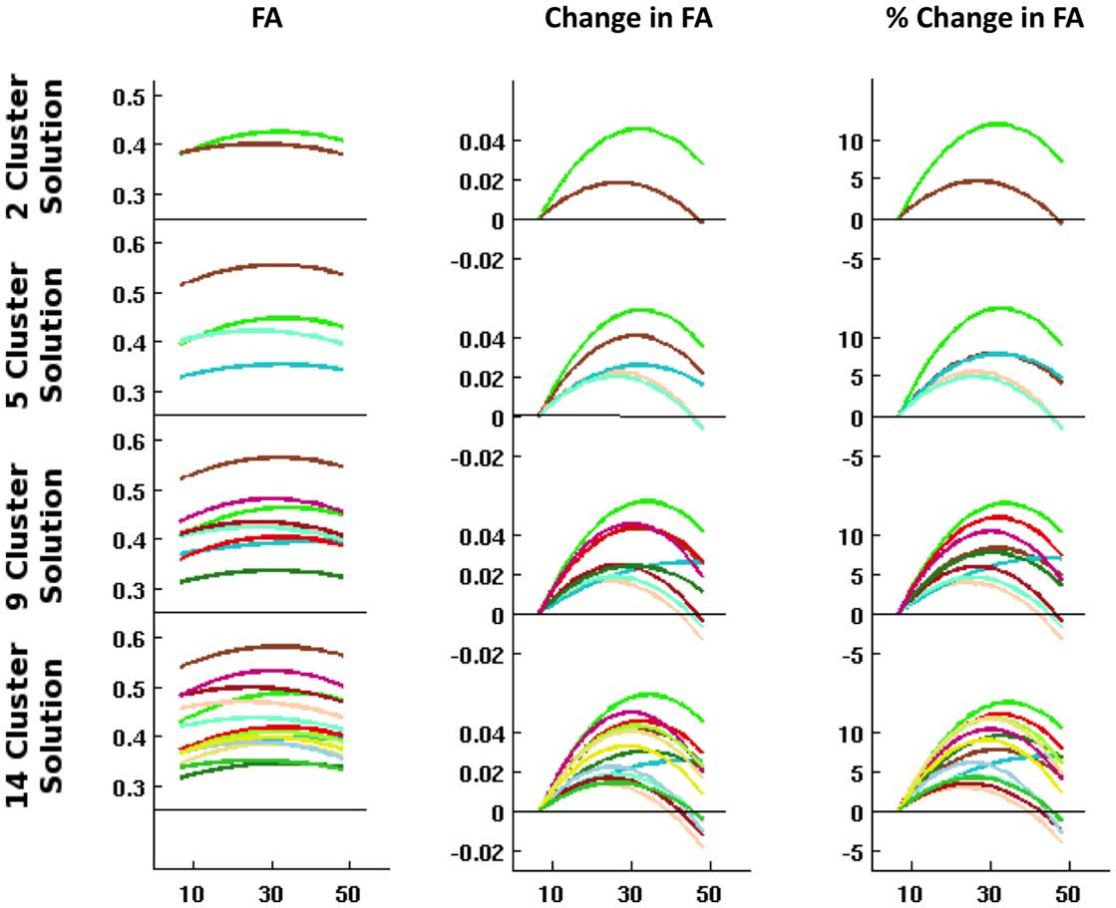
Absolute Trajectories and Relative Changes of Skeletonized Fractional Anisotropy (FA) Values. The trajectories for the 2-, 5-, 9- and 14-cluster solutions (from top to bottom) are depicted as absolute FA values (left column; ordinate shows FA absolute values), FA difference from initial trajectory value (middle column; ordinate shows relative FA change in absolute units), and percent change in FA relative to initial value (right column; ordinate shows percent change in FA). Abscissas show age in years. Initial FA trajectory value and percent change in FA were not significantly related across any of the clusters.

As expected, across all cluster solutions, age-related increases in FA were observed during childhood and adolescence. Across clusters, marked variation was noted in the magnitude of FA increases during development, even when differences in the initial FA values (i.e., FA values at age 7) were taken into account (i.e., by analyzing %-increase in FA; see Figure 2). Importantly, the F-test suggested that the quadratic fit was significantly better than the linear fit for all clusters except one, which was based in the middle and inferior cerebellar peduncles (see Figure 1B: cluster 4 in the 14-cluster solution). For the remaining clusters, examination of peak-ages obtained from the quadratic trajectories suggested that age-related decreases in FA during adulthood begin at a surprisingly early age (median peak age: 30.1 years; range: 23–34; see Table 1 for cluster trajectory characteristics).

**Table 1:** 14-Cluster Solution Fractional Anisotropy (FA) Trajectory Characteristics.

Cluster #	Cluster Components Based on ICBM DTI Atlas	Initial FA	FA at Peak	Age at Peak	Increase in FA (Initial to Peak)	% Increase in FA (Initial to Peak)	Loss in FA (Peak to Final)	% Loss in FA (Peak to Final)	Trajectory Curvature (1e-4)
	(tracts accounting for 5% or more of cluster voxels)								
1	12% Anterior limb of internal capsule R	0.43	0.49	34.7	0.059	13.67	−0.014	−2.7976	−0.7674
	11% Anterior limb of internal capsule L								
	10% Cerebral peduncle R								
	10% Cerebral peduncle L								
	8% Anterior corona radiata R								
2	81% Body of corpus callosum	0.54	0.58	31.6	0.042	7.69	−0.018	−3.1647	−0.6862
	15% Genu of corpus callosum								
3	20% Superior corona radiata R	0.46	0.47	23.1	0.013	2.90	−0.032	−6.7291	−0.5110
	19% Retrolenticular part of internal capsule R								
	18% Superior corona radiata L								
	10% Posterior limb of internal capsule R								
	10% Posterior limb of internal capsule L								
	9% Posterior corona radiata R								
4	96% Middle cerebellar peduncle	0.37	0.40	48.0	0.025	6.84	*	*	*
5	23% Anterior corona radiata L	0.42	0.44	26.2	0.018	4.34	−0.023	−5.3427	−0.4926
	22% Genu of corpus callosum								
	21% Anterior corona radiata R								
	7% Superior corona radiata L								
	6% Superior corona radiata R								
	6% Splenium of corpus callosum								
6	49% Posterior thalamic radiation R	0.48	0.50	24.6	0.016	3.40	−0.029	−5.8145	−0.5299
	44% Posterior thalamic radiation L								
7	25% Superior longitudinal fasciculus R	0.37	0.42	32.7	0.045	12.19	−0.016	−3.8303	−0.6867
	22% Superior longitudinal fasciculus L								
	10% External capsule L								
	9% External capsule R								
	9% Cingulum (cingulate gyrus) L								
	8% Cingulum (cingulate gyrus) R								
8	13% Middle cerebellar peduncle	0.32	0.35	33.6	0.030	9.47	−0.009	−2.5351	−0.4232
	9% Fornix (cres) / Stria terminalis L								
	9% External capsule L								
	7% Sagittal stratum L								
	7% External capsule R								
	6% Anterior corona radiata L								
9	39% Splenium of corpus callosum	0.48	0.53	30.1	0.050	10.30	−0.030	−5.6402	−0.9343
	11% Posterior corona radiata L								
	9% Posterior thalamic radiation R								
	9% Posterior thalamic radiation L								
	7% Retrolenticular part of internal capsule L								
	6% Superior longitudinal fasciculus L								
	6% Posterior corona radiata R								
10	21% Cingulum (hippocampus) R	0.37	0.41	31.2	0.043	11.70	−0.021	−5.0106	−0.7304
	15% Splenium of corpus callosum								
	15% Body of corpus callosum								
	12% Cingulum (hippocampus) L								
	8% External capsule L								
11	16% Splenium of corpus callosum	0.34	0.39	30.2	0.040	11.63	−0.024	−6.1696	−0.7473
	14% Superior longitudinal fasciculus L								
	12% Posterior thalamic radiation L								
	6% Sagittal stratum L								
	6% Genu of corpus callosum								
12	41% Posterior corona radiata R	0.37	0.39	25.6	0.022	6.04	−0.032	−8.3368	−0.6427
	24% Posterior corona radiata L								
	17% Superior longitudinal fasciculus R								
	11% Splenium of corpus callosum								
	6% Superior longitudinal fasciculus L								
13	17% Retrolenticular part of internal capsule L	0.37	0.40	29.0	0.033	8.91	−0.024	−6.0646	−0.6716
	10% Sagittal stratum R								
	8% Superior corona radiata L								
	8% Anterior corona radiata L								
	7% Superior corona radiata R								
	7% Posterior limb of internal capsule L								
	6% Anterior corona radiata R								
14	18% External capsule R	0.34	0.35	26.1	0.014	4.15	−0.018	−5.2006	−0.3821
	17% External capsule L								
	10% Splenium of corpus callosum								
	8% Middle cerebellar peduncle								

In the second column, cluster components are presented in terms of the relative contributions of ICBM-81 Atlas defined tracts. Columns 3–10 provide measures of age-related increases in FA during development, as well as age-related decreases during adulthood and trajectory curvature (second-order parameter from quadratic model).

Given the large number of results provided by our method, here we highlight key tracts and distinctions that illustrate the utility of this approach and potential resulting insights derived from the optimal cluster solutions (see Figures 1a and 1b). To demonstrate the ability of our method to delineate well-established tracts, as well as potential subdivisions within them, we also compared the 14-cluster solution with tracts defined by a “gold standard,” the DTI-81 Atlas [37] (see Tables 1 and 2). FA trajectory parameters for the DTI-81 Atlas tracts themselves are reported in Table S2. Unless otherwise noted, a high degree of homotopy was noted for our findings.

**Table 2:** Division of ICBM-81 DTI Atlas Tracts Across Clusters.

						PERCENTAGE OF DTI-81 ATLAS TRACT VOXELS IN CLUSTER								
ICBM DTI-81 Atlas Tract	Cluster 1	Cluster 2	Cluster 3	Cluster 4	Cluster 5	Cluster 6	Cluster 7	Cluster 8	Cluster 9	Cluster 10	Cluster 11	Cluster 12	Cluster 13	Cluster 14
**Middle cerebellar peduncle**	8	0	0	**57**	0	0	1	17	0	1	2	0	0	12
**Pontine crossing tract (a part of MCP)**	5	0	0	3	0	0	0	10	0	9	0	0	21	**51**
**Genu of corpus callosum**	0	28	0	0	**59**	0	0	1	0	1	4	0	1	5
**Body of corpus callosum**	0	**81**	1	0	7	0	0	0	1	6	1	0	2	1
**Splenium of corpus callosum**	0	0	1	0	11	1	3	2	**47**	8	7	2	3	16
**Fornix (column and body of fornix)**	0	0	1	0	20	0	0	9	0	0	2	0	0	**68**
**Corticospinal tract R**	**79**	0	0	1	0	0	0	6	0	0	2	0	1	11
**Corticospinal tract L**	**87**	0	0	0	0	0	0	1	0	0	2	0	0	10
**Medial lemniscus R**	**77**	0	0	1	0	0	0	1	0	0	7	0	1	13
**Medial lemniscus L**	**69**	0	0	5	0	0	0	14	0	0	2	0	0	10
**Inferior cerebellar peduncle R**	29	0	0	2	0	0	0	**64**	0	0	4	0	0	0
**Inferior cerebellar peduncle L**	38	0	0	6	0	0	2	**40**	0	0	7	0	4	2
**Superior cerebellar peduncle R**	**68**	0	0	1	0	0	3	28	0	0	0	0	0	0
**Superior cerebellar peduncle L**	**83**	0	0	1	0	0	0	15	0	0	0	0	0	0
**Cerebral peduncle R**	**82**	0	0	0	0	0	1	9	1	3	3	0	0	1
**Cerebral peduncle L**	**78**	0	0	0	0	0	1	12	1	1	2	0	2	2
**Anterior limb of internal capsule R**	**78**	0	0	0	0	0	0	4	1	0	0	0	1	16
**Anterior limb of internal capsule L**	**69**	0	0	0	0	0	2	7	1	0	3	0	13	7
**Posterior limb of internal capsule R**	30	0	**37**	0	0	0	0	9	0	0	2	0	5	16
**Posterior limb of internal capsule L**	22	0	**37**	0	0	0	0	5	6	0	5	0	17	9
**Retrolenticular part of internal capsule R**	0	0	**77**	0	0	0	0	5	6	0	0	0	1	11
**Retrolenticular part of internal capsule L**	0	0	9	0	1	0	0	12	27	0	4	0	**46**	1
**Anterior corona radiata R**	24	0	0	0	**57**	0	4	4	0	1	0	0	7	3
**Anterior corona radiata L**	14	0	0	0	**59**	0	4	10	0	0	1	0	9	1
**Superior corona radiata R**	5	5	**48**	0	21	0	1	1	0	0	1	0	11	8
**Superior corona radiata L**	8	1	**43**	0	24	0	1	2	0	3	1	0	13	5
**Posterior corona radiata R**	0	0	**35**	0	17	1	0	0	21	0	2	21	1	2
**Posterior corona radiata L**	0	0	14	0	20	1	0	2	**43**	4	2	13	1	1
**Posterior thalamic radiation R**	0	0	1	0	4	**58**	1	0	24	1	0	0	3	8
**Posterior thalamic radiation L**	0	0	0	0	0	**55**	1	3	23	2	12	0	3	1
**Sagittal stratum R**	0	0	6	0	0	6	28	18	0	0	9	0	**33**	0
**Sagittal stratum L**	0	0	0	0	0	3	13	**50**	0	11	14	0	5	3
**External capsule R**	0	0	0	0	0	0	25	18	0	0	1	0	3	**52**
**External capsule L**	0	0	0	0	0	0	26	21	0	7	0	0	3	**43**
**Cingulum (cingulate gyrus) R**	0	0	0	0	2	0	**70**	8	8	2	8	0	1	0
**Cingulum (cingulate gyrus) L**	1	1	0	0	2	0	**79**	0	5	1	9	0	1	0
**Cingulum (hippocampus) R**	0	0	0	0	0	0	0	11	0	**82**	0	0	6	0
**Cingulum (hippocampus) L**	15	0	0	0	0	0	0	8	25	**53**	0	0	0	0
**Fornix (cres) / Stria terminalis R**	1	0	0	0	9	0	0	**70**	0	1	1	0	2	16
**Fornix (cres) / Stria terminalis L**	0	0	0	0	5	0	1	**91**	0	0	0	0	1	2
**Superior longitudinal fasciculus R**	0	0	8	0	0	0	**63**	1	9	1	1	5	0	13
**Superior longitudinal fasciculus L**	0	0	5	0	0	0	**59**	1	13	3	11	2	6	1
**Superior fronto-occipital fasciculus R**	27	0	0	0	23	0	0	6	0	0	0	0	0	**44**
**Superior fronto-occipital fasciculus ) L**	8	0	0	0	8	0	0	**56**	0	0	6	0	10	10
**Uncinate fasciculus R**	0	0	0	0	0	0	**44**	3	0	19	0	0	19	15
**Uncinate fasciculus L**	0	0	0	0	0	0	30	6	0	4	0	0	**39**	21
**Tapetum R**	0	0	0	0	0	0	0	0	**100**	0	0	0	0	0
**Tapetum L**	0	0	0	0	0	0	0	0	**100**	0	0	0	0	0

Atlas-defined tracts are presented in terms of their %voxels located in each of the clusters from the 14-cluster solution. For each tract, the bold-faced number indicates the cluster containing the largest percentage of that tract's voxels.

### Specific Findings

#### Corpus Callosum

First, we draw attention to the corpus callosum (CC), the primary conduit for interhemispheric communication between the cerebral hemispheres. Despite the lack of *a priori* specification or bias, cluster analyses delineated three well-established divisions within the CC based upon differences in their developmental trajectories – namely, the splenium (posterior), the genu (anterior) and the body (intermediate) [38]. The splenium differentiated from the remainder of the CC as early as the 2-cluster solution. The three CC divisions fully separated from one another by the 5-cluster solution. Among the three divisions, the splenium, which supports interhemispheric transfer between visual cortex, exhibited the largest age-related increases in FA, more than doubling that observed in the genu and nearby prefrontal areas (10.3% vs. 4.3%); the body of the CC fell between the two other divisions (7.2%).

Of note, with respect to absolute FA values, the CC body exhibited the highest initial and peak FA, and the genu the lowest. These differences are consistent with prior findings regarding fiber size and myelination [39]. Specifically, the CC body, which supports communication among motor and somatosensory regions, has the greatest preponderance of gigantic and large axons. In contrast, the genu, which is intimately associated with prefrontal cortex, is populated primarily by small and medium myelinated axons, and contains the highest fraction of unmyelinated neurons among callosal regions [39].

#### Cerebral Peduncles, Internal Capsule and Cingulum Bundle

Across cluster solutions, the cerebral peduncles consistently clustered with the anterior limb of the internal capsule. The two combined exhibited the greatest increases in FA during development (indexed with both absolute difference and %-increase). They also ranked among the lowest with respect to measures of loss during adulthood, suggesting relative preservation of their integrity with age. The strength of the relationship between voxels within the anterior limb of the internal capsule and the cerebral peduncles is not surprising, given that the fronto-pontine tracts, which project from the frontal cortex to the pons, travel through the anterior limb of the internal capsule [40], [41]. Interestingly, the cingulum bundle and portions of the superior longitudinal fasciculus do not differentiate from the cerebral peduncle-internal capsule tracts until the 9-cluster solution. This underlines a remarkable degree of similarity among these tracts with respect to their developmental trajectories.

We note that distinctions between the anterior and posterior limbs of the internal capsule were prominent across all cluster solutions – likely reflecting their different functions. The anterior limb conveys the fronto-pontine and thalamo-cortical pathways, both of which are critical to information transfer with the prefrontal lobes [40], [41]. In contrast, the posterior limb transmits sensory and motor information [42], [43], [44]. Consistent with this distinction, tracts such as the optic radiations and the corticospinal tracts (above the level of the cerebral peduncles) were associated with the posterior limb rather than with the anterior limb. Finally, with respect to the corticospinal tracts, we note that our analyses consistently differentiated portions above the level of the cerebral peduncles from those within (see Figure 1a).

### General Linear Model Based Voxel-wise Results

**Figure 3:**
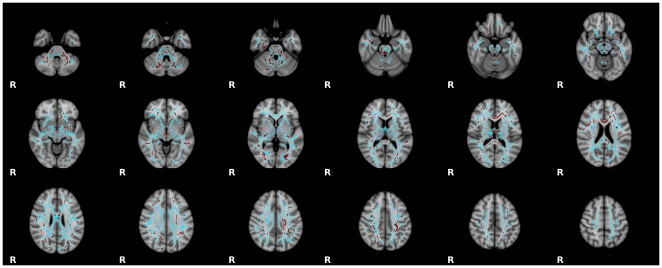
Model-Based Trajectory Analyses. Permutation-based non-parametric testing, using a general linear model containing linear and quadratic terms, revealed significant age-related changes in skeletonized white matter throughout the brain (*p*<0.05, corrected). We employed an F-test to determine optimal fit (linear vs. quadratic). Tracts for which the optimal fit was the inverted quadratic (‘U-shaped’) developmental trajectory are shown in blue, and those for which the optimal fit was the linear trajectory are shown in red.

Although not the primary focus of the proposed work, we carried out traditional multiple regression-based analyses of voxel-wise increases in FA. For these analyses we used linear and quadratic models. Linear models revealed overall age-related increases in FA for most WM tracts (p<0.05, corrected). Quadratic models, which detected curvilinear trends associated with age-related decreases in adulthood, were also significant (p<0.05, corrected). F-test comparisons revealed a significantly better fit for the quadratic models, further highlighting the non-linearity of age-related changes in FA. No significant linear negative effects of age were noted (see Figure 3).

## Discussion

Cluster analyses of FA of skeletonized white matter tracts successfully differentiated the major tracts and subdivisions, based on their developmental trajectories in a cross-sectional DTI sample spanning ages 7–48 years. While nearly all tracts exhibited quadratic trajectories for FA, they differed with respect to the magnitude of increases in white matter during childhood and adolescence, as well as in the rates and extent of decreases during middle adulthood. These findings recapitulate those of prior DTI and histological studies [37], [45], thus validating our proposed analytical approach. Additionally, they provide new insights regarding the nature of the different developmental trajectories and age-related compromises associated with distinct white matter tracts and subdivisions.

Despite its initial development in the mid 1980's [46], [47], the application of DTI to the examination of brain development has lagged behind other anatomical and functional MRI approaches. Recent years have witnessed numerous methodological innovations in the acquisition, reconstruction and processing of diffusion data with the goal of overcoming imaging artifacts that hamper tractography-based approaches [48], [49], [50], [51], [52]. Our findings suggest that a wealth of information can be extracted with existing technologies and datasets. In the present work, cluster analysis based on developmental trajectories not only captured well-established structural distinctions within tracts such as the corpus callosum and internal capsule, but highlighted developmental similarities between tracts such as the anterior limb of the internal capsule and the cerebral peduncles, the posterior limb of the internal capsule and the optic radiations, and the cingulum bundle and cerebral peduncles. While the commonalities among such tracts may represent sources of error for tractography approaches, they become rich sources of information for model-free analyses. Equally exciting is the ability of our approach to reveal consistent distinctions within a tract across all or most cluster solutions. Examples of structures that divided as early as the 2-cluster solution and remained separate include: the splenium, which separated from the remainder of the CC, the anterior and posterior limbs of the internal capsule, and the corticospinal tract, which divided at the level of the cerebral peduncles. An important advantage of our approach over tractography approaches is its independence from *a priori* expectations. As such, it lends itself well to both hypothesis generation and replication studies.

The prominence of quadratic trajectories in our sample is particularly noteworthy, as the findings do not simply reflect curvilinear patterns during early development. Rather, they appear to reflect decreases in white matter integrity that are estimated to begin in the 3^rd^ decade of life for nearly half the tracts examined. A striking aspect of our results was the young age at which maximal FA was estimated to be reached, which presumably is also when age-related decline in FA can be estimated to begin. Across clusters, peak ages based on quadratic fits varied between 23 and 34 years old, and estimated absolute loss by age 48 reached between 5 and 8% for several clusters. This age range surprised us, as WM volume loss has generally been documented to occur at or after age 40 [53]. However, Salat et al. [54] noted that FA and white matter volume are not strongly related prior to age 40. These authors also suggested that FA changes related to aging are “due to microstructural alterations that are antecedent to WM hyperintensities or volume loss commonly measured in MR studies” (p. 45, [54]). It is also possible that our ability to detect age-related changes was enhanced by pooling FA across voxels within structural units in a data-driven manner (i.e., tracts, sub-regions of tracts, sets of tracts revealed by the cluster analysis). This may have avoided potential false negatives due to noise associated with averaging across voxels that, while geographically members of the same large-scale anatomical tract, exhibit differential developmental trajectories. Replication of these findings is clearly warranted, ideally with a broader range of ages and denser sampling at the age extremes to avoid potential artifacts. If replicated, our findings would suggest that correlates of brain aging can be detected earlier than previously thought, with implications for the development of potential interventions.

Intriguingly, well-established subdivisions of the corpus callosum were delineated by cluster analyses. Specifically, the genu, which supports interhemispheric transmission among frontal regions, showed a more gradual pattern of increases in FA [55], [56] and earlier age-related losses, relative to the splenium, which supports transmission in posterior sensory regions. These findings suggest both the primacy of myelination in tracts supporting sensory function, as well as their preservation. Across clusters, it was subcortical and brainstem-based tracts, along with the splenium, that tended to exhibit the earliest and most preserved increases in FA. In combination, these findings are consistent with the larger developmental and aging literatures, and appear to reflect ontogenic and phylogenic providence and fates in terms of lifespan development. Specifically, phylogenetically primitive sensorimotor brain structures tend to show the most rapid development and greatest preservation, while more phylogenetically advanced structures, such as prefrontal cortex, tend to show slower development and faster declines, suggesting a first-in-last-out pattern of development across the life span [55], [56], [57].

While model-free data-mining is extensively used in the biological community, particularly in genetics, it is only recently gaining acceptance in neuroimaging. In particular, independent component analysis (ICA) has played a major role in revealing the brain's functional architecture, and more recently, its structural architecture [57]. Cluster analysis has also emerged as powerful tool for exploring functional and structural datasets, with a number of recent successes [58], [59], [60], [61], [62]. Graph theory and machine-learning approaches to data-mining have also begun to emerge [63], [64], [65], [66], [67], further increasing the array of data-mining tools available to neuroscientists – each with unique strengths and limitations. Although this diversity of options is gratifying, it also calls for rigorous comparisons of the relative advantages and disadvantages of data-mining approaches to minimize the introduction of false positive and negatives into this rapidly developing literature.

We note a number of methodological considerations and limitations related to our results - particularly the limitations inherent to our sample. Our age range did not capture the developmentally active period prior to age 7 years, nor did it include adulthood beyond the fifth decade, when the greatest losses of structural integrity in the brain occur. The limited age range may explain the relatively low complexity of trajectories identified, which mostly tended to be quadratic. We chose to avoid higher-order models (e.g., cubic) for characterizing trajectories, to prevent artifactual fits that can arise at the extremes of smaller samples, such as ours. A larger sample including individuals spanning the entire lifespan will likely reveal more complex developmental trajectories. Greater sampling densities (i.e., greater number of participants per age bracket) in future cross-sectional studies will bolster efforts to define age-based trajectories with a higher degree of fidelity. Even more important will be the incorporation of longitudinal designs. Future work may also take advantage of the proposed approach to allow exploration of developmental trajectories in clinical populations, without assumptions based upon neurotypical populations (e.g., tract definition, developmental relationships between tracts).

Additionally, the resolution of our diffusion data was relatively low (3-mm isotropic), and we did not take advantage of more advanced acquisition and/or reconstruction strategies (e.g., HARDI, Q-BALL, DSI) [52], [68], [69], [70]. Nonetheless, a strength of the present approach is its applicability in datasets that are otherwise poorly suited for tractography (i.e., those containing few diffusion directions). However, higher resolution and higher quality diffusion datasets should allow for more fine-grained parcellations of white matter tracts. Future work needs to consider the relative merits of carrying out cluster analyses at the full brain level, as in the present work, compared to more limited examinations designed to yield greater differentiation in complex tracts of interest. Finally, the present work was carried out using skeletonized FA generated with the FSL TBSS processing pipeline, which limits white matter to voxels thresholded at FA>0.2. This analysis therefore excludes gray matter and CSF, as well as portions of white matter with less robust diffusion signal. Future work should explore the utility of cluster analysis based approaches in alternative DTI estimation processing pipelines.

In conclusion, data-driven clustering methods applied to DTI FA in a cross-sectional sample extending from childhood to mid-adulthood revealed distinct developmental trajectories in major white matter tracts and their subdivisions. Independent replication of these results will provide a powerful means of generating and testing novel hypotheses regarding structural connectivity in the human brain.

## Supporting Information

Figure S1 **Evolution of Cluster Solutions.** *Panel A (Skeletonized Fractional Anisotropy [FA])*: K-means cluster analysis was employed to group and differentiate white matter voxels based on their cross-sectional age-related fractional anisotropy (FA) trajectory across individuals aged 7–48 years. Skeletonized FA for the first five cluster solutions are depicted in each column in MNI space, revealing a high degree of stability across solutions. In each column (solution), each color represents a distinct cluster. *Panel B (Trajectories and Atlas-Based Projections)*: The first five cluster solutions are depicted in rows. Scatter plots for each cluster in a given solution show the data points included in each cluster and their mean trajectories. All trajectories are baselined with respect to the initial trajectory value to facilitate visual comparison. Age in years is shown on the abscissas. The middle graph (solid black line) in the top row shows the Davies-Bouldin cluster validation index (DBI) for determining optimal clustering solutions. Local minima were detected for the 5-, 9-, and 14-cluster solutions. The trajectories for all clusters in each solution, set to initial baseline, are shown in the second column from the right. To facilitate visualization, the right-most column provides an ICBM-81 atlas-based tract projection, with each tract color-coded based upon the dominant cluster to which its voxels were assigned.
(TIF)

Table S1 **Evaluation of tract continuity within 5, 9, and 14-cluster solutions.** For each solution, we list the following for each cluster: 1) % of voxels contained in the largest continuous tract within the cluster, and 2) the percentages of voxels that are part of large continuous tracts within the cluster (i.e., tract size> = 500 voxels). Measures are reported separately for each hemisphere (left, right) and the whole brain (whole).
(XLS)

Table S2 **Fractional Anisotropy (FA) Trajectory Characteristics for ICBM-81 DTI Atlas Tracts.** Measures of age-related increases in FA during development are provided for each of the ICBM-81 DTI Atlas *Tracts*, as well as age-related decreases during adulthood and trajectory curvature (second-order parameter from quadratic model).
(XLS)
