# Supplementary figures and images for: Differential Development of Human Brain White Matter Tracts
Source: PLoS One. 2011 Aug 31;6(8):e23437. doi: 10.1371/journal.pone.0023437 (PMC3166135; doi:10.1371/journal.pone.0023437)

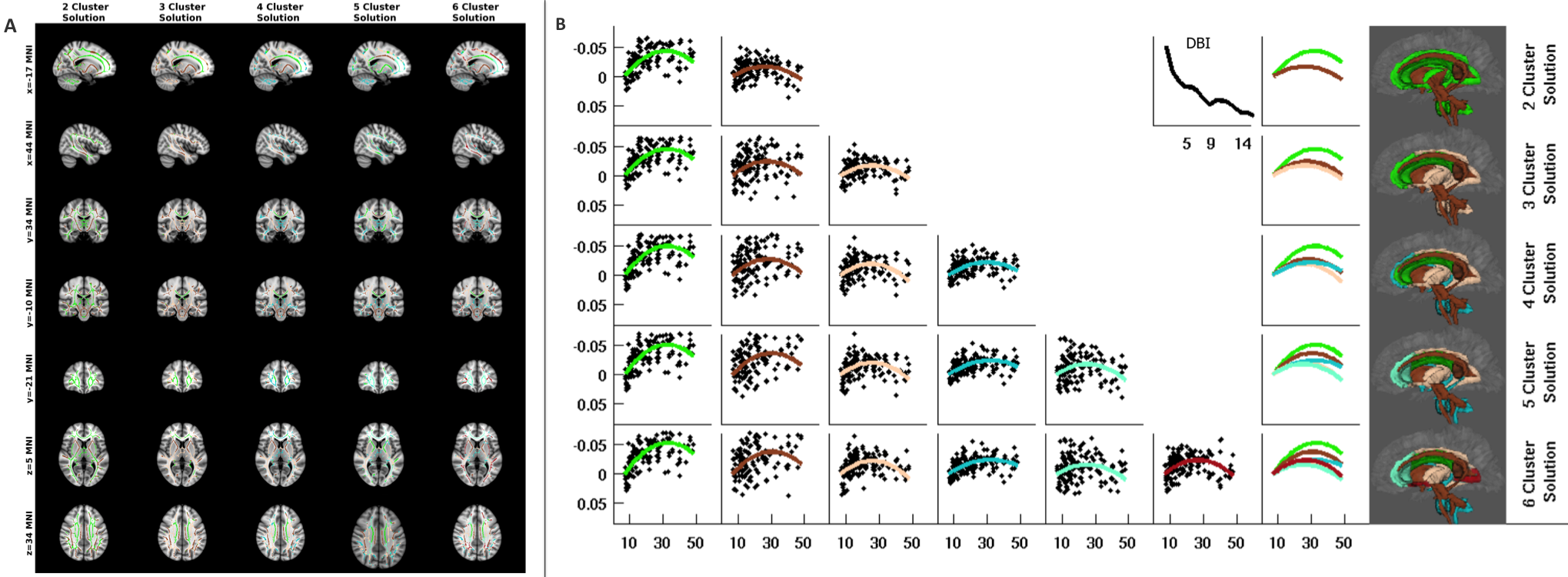

Supplement: Figure S1 — Evolution of Cluster Solutions. Panel A (Skeletonized Fractional Anisotropy [FA]): K-means cluster analysis was employed to group and differentiate white matter voxels based on their cross-sectional age-related fractional anisotropy (FA) trajectory across individuals aged 7–48 years. Skeletonized FA for the first five cluster solutions are depicted in each column in MNI space, revealing a high degree of stability across solutions. In each column (solution), each color represents a distinct cluster. Panel B (Trajectories and Atlas-Based Projections): The first five cluster solutions are depicted in rows. Scatter plots for each cluster in a given solution show the data points included in each cluster and their mean trajectories. All trajectories are baselined with respect to the initial trajectory value to facilitate visual comparison. Age in years is shown on the abscissas. The middle graph (solid black line) in the top row shows the Davies-Bouldin cluster validation index (DBI) for determining optimal clustering solutions. Local minima were detected for the 5-, 9-, and 14-cluster solutions. The trajectories for all clusters in each solution, set to initial baseline, are shown in the second column from the right. To facilitate visualization, the right-most column provides an ICBM-81 atlas-based tract projection, with each tract color-coded based upon the dominant cluster to which its voxels were assigned. (TIF) [file pone.0023437.s001.tif]
